# Different dissipation potential and dietary risk assessment of tristyrylphenol ethoxylates in cowpea ecosystem in China

**DOI:** 10.3389/fnut.2022.1036025

**Published:** 2022-10-19

**Authors:** Minjie Li, Qi Wang, Xiaohui Li, Ning Yue, Maojun Jin, Lufei Zheng, Jing Wang, Fen Jin

**Affiliations:** Key Laboratory of Agro-Product Quality and Safety, Institute of Quality Standards and Testing Technology for Agro-Products, Chinese Academy of Agricultural Sciences, Beijing, China

**Keywords:** tristyrylphenol ethoxylates, cowpea, soil, dissipation, risk assessment

## Abstract

Tristyrylphenol ethoxylates (TSPEOn) are widely used as inert ingredients in pesticide formulations in the world. However, the information on the dissipation behavior of different homologs TSPEOn in agro-products is lacking. To investigate the dissipation behavior of TSPEOn, a cowpea field experiment treated with TSPEOn at different doses was carried out in Guangdong province, China. Different 24 TSPEO homologs were all detected in cowpea from the field terminal residue experiments, and the total concentrations of TSPEO homologs in cowpea were 40.0–1,374 μg/kg. The dissipation half-lives of 24 TSPEO homologs in soil were 1.51–2.35 times longer than those in cowpea. The long-chain homologs TSPEOn were dissipated faster than the short-chain homologs TSPEOn, suggesting a homolog-specific degradation of the TSPEOn in the cowpea ecosystem. The characteristic bimodal profiles of TSPEOn (*n* = 6–29) differing from that of the commercial TSPEOn were observed in the cowpea terminal residues experiment, indicating that the long-chain TSPEOn would degrade to short-chain TSPEOn in cowpea and soil. The acute and chronic dietary exposure risks of ΣTSPEOn in cowpea are within acceptable margins for human consumption across different ages and genders. But the health risks to children should be noticed in future.

## Introduction

**FIGURE 1:**
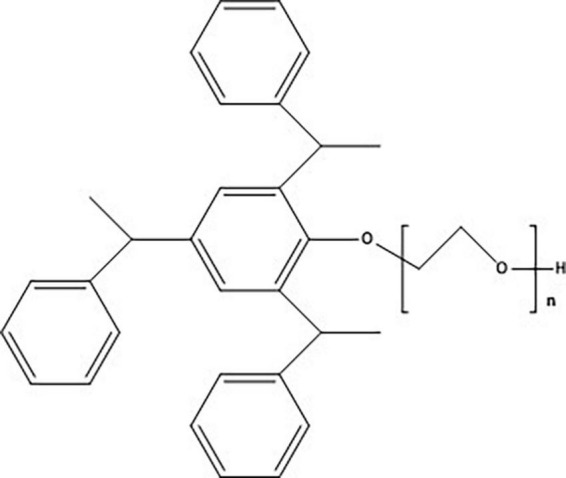
Chemical structure of tristyrylphenol ethoxylates (TSPEOn) (*n* = 1–33).

Tristyrylphenol ethoxylates (TSPEOn) are important nonionic surfactants which are widely used in pesticide formulations to enhance the penetration and spread of the active ingredient. As the nonionic surfactant, TSPEOn was second only to alkylphenol ethoxylates (APEOn) in China (1). A typical TSPEOn surfactant formulation is comprised of tristyrene with an average of 16 ethoxylate (EO) units, usually within the range of 1 to 33 ethoxylate units as depicted in Figure 1 (2, 3). Studies have shown that TSPEOn had moderate acute toxicity, subchronic toxicity, thyroid, and liver toxicity in mammals (4, 5). Furthermore, its degradation intermediates, styrenated phenols were demonstrated to have acute toxicity or estrogenic activity in *Pseudokirchneriella subcapitata* and *Oryzias latipes* (6–8). Considering the toxicity and the large production volumes, the United States Environmental Protection Agency has set a TSPEOn limit of no more than 15% in pesticide formulations in 2010 (4). However, concern about its residue and environmental behavior continue to this day, such information is currently lacking.

Previous studies had shown that relatively high concentrations of TSPEOn were detected in the agricultural ecosystem, such as cherries, peaches, and kiwifruit (1). Additionally, the dissipation behavior of TSPEOn was reported in lettuce under greenhouse and field conditions with half-lives of 2.18–5.36 and 1.82–5.52 days, respectively. TSPEOn were relatively persistent in the field. It can be concluded that the cultivation system and plant type jointly affect the absorption and degradation of TSPEOn (9, 10). Cowpea [Vigna unguiculata (L.) Walp.] is an ideal food for diabetic due to its phospholipid can promote insulin secretion and participate in glucose metabolism, which is widely cultivated in the tropical and subtropical region of Asia. However, cowpea is susceptible to a variety of diseases and insect infestations, such as aphids, thrips, cowpea weevil, and liriomyza (11–15). Pesticide application is a probable major source of TSPEO residues during cowpea cultivation (11, 16–18). Further research is needed to study the potential different dissipation behavior of TSPEOn by cowpea growing in terms of public health and food safety.

In this study, a cowpea field experiment was carried out in Guangdong province, the main region of cowpea production in China, which was treated with TSPEOn at different doses. Different 24 tristyrylphenol ethoxylate homologs were all analyzed in cowpea from the field experiments to shed light on the dissipation rates and distribution profiles of different TSPEO homologs in cowpea. The acute and chronic dietary exposure risks of TSPEOn in cowpea for different subgroups (age and gender) based on supervised field trial data and relevant toxicological parameters were also assessed. The results obtained in this study have important implications in understanding the residue fate of TSPEOn.

## Materials and methods

### Reagents and chemicals

The standard of Technical TSPEO16 (a mixture of TSPEOn with an average of 16 EO units) was purchased from Jiangsu Zhongshan Chemical Co., Ltd., (Nanjing, China) and purified by using preparative liquid chromatography (LC) as described in our earlier study (19). Ultrapure water (18.2 MΩ⋅cm) was prepared by Milli-Q purification system (Millipore, Bedford, MA, USA). Octadecyl (C18) and primary secondary amine (PSA) sorbents were purchased from Bonna-Agela Technologies, Ltd., (Tianjin, China). Multiwalled carbon nanotubes (MWCNTs) were obtained from Nanjing XFNANO Materials Technologies (Nanjing, China). Acetonitrile (≥ 99.95%) was liquid chromatography-mass spectrometry (LC-MS) grade (Thermo Fisher Scientific, Waltham, MA, USA). Anhydrous magnesium sulfate and sodium chloride were analytical grade (Sinopharm Chemical Reagent Company, Beijing, China).

### Field trails and sampling

Field trials of cowpea were designed under open conditions according to the Guideline for testing pesticide residues in crops (NY/T 788-2018) and the Standard operating procedures on pesticide registration residue field trials (20). For the field dissipation experiments, the emulsifier 601 (Technical TSPEO16) was diluted with water (500-fold dilution) and sprayed on the cowpea and bare soil at a dose of 2,250 g/ha during the vegetative period. A separate plot with the no-TSPEOn application was used as a control. Cowpea planting density and fertilization management in the experimental field were designed, according to the conditions of local planting. The area of each plot was 15 m^2^. Representative 2 kg cowpea and soil samples were collected randomly from each plot at 2 h, 1 d, 3 d, 5 d, 7 d, 10 d, 14 d, and 21 d after spraying. Both the cowpea and soil samples were stored in plastic bags with proper labels before being transferred to the laboratory.

For the terminal residue experiments, the emulsifier 601 was applied at dosage of 225 g/ha and 450 g/ha, respectively. Two and three applications were made with an interval of 5 d. Representative 2 kg cowpea and soil samples were collected separately from each plot at 5, 7, 10, 14, and 21 d after the last application. The mature cowpea samples were collected from the top, middle, and bottom of the shelf from each plot. All cowpea samples were cut into small pieces, homogenized and stored at −20°C until analysis. All soil samples were collected from 0 to 15 cm of the layer, dried at room temperature, ground to a powder using an electric grinder and sifted through a 2-mm sieve. All samples were packed in seal aluminum foil bags, and then stored at –20°C until analysis.

### Instrument condition

Tristyrylphenol ethoxylates (TSPEOn) analysis was performed by high performance liquid chromatography-tandem mass spectrometry (HPLC-MS/MS) according to our previous study (1). Shimadzu Triple Quadrupole LCMS-8050 system (Shimadzu, Kyoto, Japan) equipped with a Xbridge C18 (2.1 × 50 mm, 5 μm, Waters, Milford, MA, USA) precolumn and a Nova-Pak Silica (2.1 × 150 mm, 4 μm, Waters, Milford, MA, USA) column were used to separate the different homolog TSPEOn. The flow was kept at 0.30 mL/min. The mobile phases were 2 mM ammonium acetate water (A) and acetonitrile (B), and the gradient elution program was as follows: mobile phase B was ramped from 95 to 88% over 5 min, varied from 88 to 80% over 5.5 min, held at 80% for 2.0 min, and then increased to 95% over 0.5 min, thereby maintaining initial chromatographic condition within 7 min. The column temperature was maintained at 40°C. The injection volume was 2 μL.

Mass spectrometry (MS/MS) analysis was accomplished using a tandem quadrupole mass spectrometer (LCMS-8050, Shimadzu, Kyoto, Japan) in time programmed multiple-reaction monitoring mode in positive mode. The source parameters were optimized and performed as follows: the ion source temperature (TEM) was 450°C. The base ions were the ammonium adduct ions [(M + NH_4_)^+^ or (M + 2NH_4_)^2+^]. All the MS parameters were listed in Supplementary Table 1 in supporting information. The LabSolutions software was used to acquire and analyze the data (version 5.82, Shimadzu).

### Sample preparation

The 10 g homogenized samples (cowpea and soil) were weighed into a 50-mL polypropylene centrifuge tube with a screw cap. To this, 10 mL ultrapure water (only to soil) and 10 mL acetonitrile were subsequently added. The sample tubes were vigorously vortexed for 1 min, and then ultrasound for 10 min. After that, 1 g sodium chloride and 4 g anhydrous magnesium sulfate were added, and the tube was vortexed for another 1 min and then centrifuged at 6,000 rpm for 5 min. 1 mL supernatant was transferred into a 10-mL centrifuge tube containing different purifying agents (150 mg anhydrous magnesium sulfate, and 5 mg MWCNTs for cowpea extraction and 5 mg MWCNTs, 25 mg PSA, and 25 mg C18 for soil extraction). After vertexing for 1 min, the tube was centrifuged at 10,000 rpm for 5 min. Finally, the resulting supernatant was filtered into an autosampler vial through a 0.22-μm membrane (Bonna-Agela Technologies Inc., Tianjin, China) for HPLC-MS/MS analysis.

### Method validation

The method validation results for TSPEOn in cowpea are shown in Supplementary Table 2. Recovery experiments were performed to evaluate the accuracy and precision of the method. Five replicates of spiked blank samples at three spiking levels were prepared. The recoveries of all the TSPEO homologs (*n* = 6–29) in cowpea ranged from 79.7 to 120%, with RSDs of 0.70–20.1%. The linearities of all the TSPEO homologs (*n* = 6–29) were evaluated by analyzing matrix-matched standard solutions, and the correlation coefficients (R^2^) were higher than 0.990. The limits of detection (LODs) and the limits of quantification (LOQs) were determined based on the signal-to-noise ratios of 3 and the lowest spiked concentration of each analyte, respectively. The LODs and LOQs for the homologs of TSPEOn were 0.001–0.14 and 0.06–5.13 μg/kg, respectively. The method validation results for TSPEOn in soil were listed in our previous research (19). The recoveries and RSDs ranged from 64.2 to 113% and 1.30 to 17.3%, respectively.

### Data processing and statistical analysis

The dissipation kinetics of all 24 TSPEO homologs in cowpea and soil were estimated according to the pseudo first-order dynamics equation:


(1) $$C_{t}=C_{0}\times e\,x\,p^{-k\,t}$$

where *C*_0_ (μg/kg) and *C*_*t*_ (μg/kg) indicate the concentrations of TSPEO homologs and ΣTSPEOn at time 0 (d) and time t (d), *k* is the dissipation rate constant. The half-life (T_1/2_) was calculated from *k* by using the equation:


(2) $$T_{1/2}=l\,n\,2/k$$

The acute dietary intake risk (aHI) was estimated based on the following equations (10, 21).


(3) N⁢E⁢S⁢T⁢I=H⁢R×L⁢P/b⁢w

(4) $$a\,H\,I=\frac{N\,E\,S\,T\,I}{A\,R\,f\,D}\times 100\%$$

where NESTI is the national estimated short-term intake. HR is the highest residue concentration (μg/kg), which is obtained on the highest residue level of the terminal residue experiments. LP is the large portion consumption of cowpea (dark-colored vegetables instead) for the consumers (97.5th percentile of eaters, g/day person), and bw is the mean body weight, which is shown in Supplementary Table 3 (11). In this study, the population was divided into eight groups according to age and gender: child (≤ 11 years), youngster (12–18 years), adult (18–60 years), and elder (> 60 years) for both male and female. The consumption data of dark-colored vegetables was used instead in the dietary risk assessment, when the cowpea consumption data were unavailable. ARfD is the acute reference dose (1.67 mg/kg/d), which was determined using the lowest observed adverse effect level of 500 mg/kg/d and an uncertainty factor of 300 (4, 22).

The chronic dietary intake risk (hazard quotient, HQ) was estimated based on the following equations (10, 21).


(5) N⁢E⁢D⁢I=S⁢T⁢M⁢R×F/b⁢w

(6) $$H\,Q=\frac{N\,E\,D\,I}{A\,D\,I}\times 100\%$$

where NEDI is the national estimated daily intake. STMR is the median residue in the terminal residue experiments (μg/kg). F is the mean daily consumption of cowpea (dark-colored vegetables instead, g/day person), as shown in Supplementary Table 3 (11), ADI is the acceptable daily intake (0.5 mg/kg/d) calculated using the no observed adverse effect level of 50 mg/kg/d and an uncertainty factor of 100 (4, 22).

## Results and discussion

### Dissipation of homolog tristyrylphenol ethoxylates (n = 6–29) in cowpea system

**FIGURE 2:**
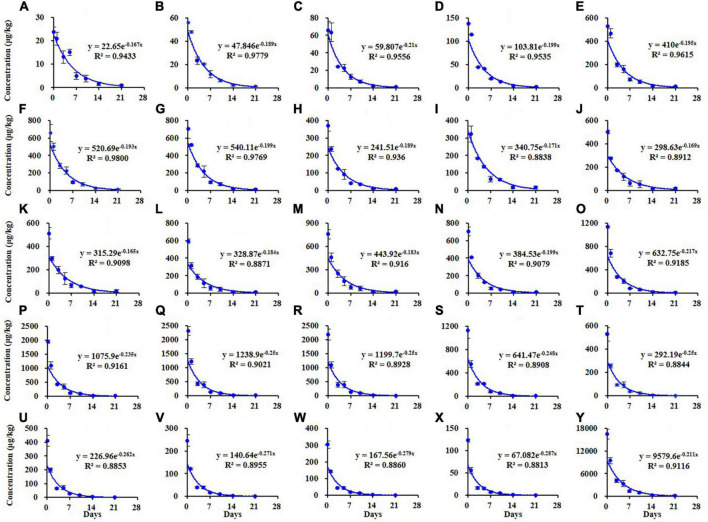
Dissipation kinetics curves of different tristyrylphenol ethoxylates (TSPEOn) [*n* = 6–29, **(A–X)**] homologs and ΣTSPEOn **(Y)** in cowpea in China.

The dissipation kinetics curves of different homolog TSPEOn (*n* = 6–29) and ΣTSPEOn in cowpea were shown in Figure 2. The initial concentrations of TSPEOn (*n* = 6–29) and ΣTSPEOn deposited on cowpea samples were 23.9–2,316 μg/kg (Figures 2A–X) and 16,506 μg/kg (Figure 2Y) at 2 h after TSPEOn treatment, respectively. After 21 d, 96.1–99.8% of the initial residues of TSPEOn (*n* = 6–29) were dissipated. The dissipation half-lives of homolog TSPEOn (*n* = 6–29) and ΣTSPEOn were found to be slightly varied from 2.42 to 4.20 d, which were comparable to those in lettuce (1.82–4.34 d) and cucumber (1.80–4.30 d) in the previous studies (9, 10), indicating that all the homolog TSPEOn (*n* = 6–29) could be dissipated fast in these vegetables.

**FIGURE 3:**
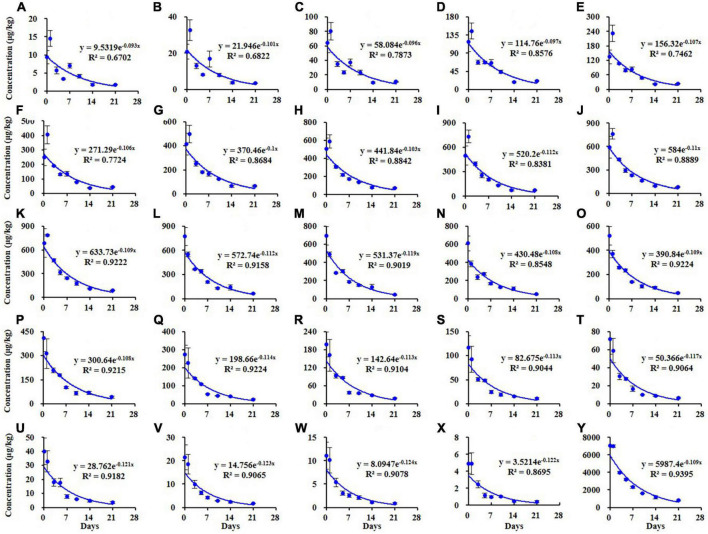
Dissipation kinetics curves of different tristyrylphenol ethoxylates (TSPEOn) [*n* = 6–29, **(A–X)**] homologs and ΣTSPEOn **(Y)** in soil in China.

**FIGURE 4:**
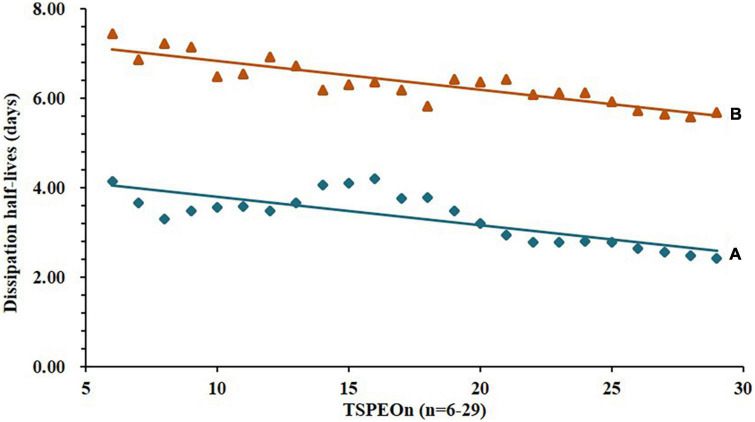
Relationship between dissipation half-lives and the different homolog tristyrylphenol ethoxylates (TSPEOn) (*n* = 6–29) in cowpea and soil. **(A)** TSPEOn half-lives in cowpea, slope = –0.0632, *r*^2^ = 0.6247; **(B)** TSPEOn half-lives in soil, slope = –0.0644, *r*^2^ = 0.7937.

Similar results were observed in the soil as shown in Figure 3. The dissipation trends of all TSPEOn (*n* = 6–29) and ΣTSPEOn followed pseudo first-order kinetics. After 21 d, the dissipation rates of homolog TSPEOn (*n* = 6–29) and ΣTSPEOn can reach 85.3–93.9% in soil, which were slightly lower than those in cowpea. The variety of dissipation rates of homolog TSPEOn (*n* = 6–29) in cowpea and soil might be related to several factors, including log Kow, climatic conditions, photo-degradation, microorganism biodegradation, preferential absorption, and character of soil (23–30). According to the length of ethoxylate chain, the TSPEOn has been divided into two groups, namely short-chain TSPEOn (*n* ≤ 16) and long-chain TSPEOn (*n* > 16) in this study. From Figures 2,  3, it was found that the dissipation half-lives of short-chain TSPEOn (*n* ≤ 16) were a little bit higher than those of long-chain TSPEOn (*n* > 16) in cowpea and soil. A regression analysis between the dissipation half-lives and the different homolog TSPEOn (*n* = 6–29) in cowpea and soil was conducted in Figures 4A,B. It was found that the dissipation half-lives of the homolog TSPEOn (*n* = 6–29) were significantly decreased with the increasing EO unites in TSPEOn structure in cowpea and soil, indicating that the length of EO chain would be an essential factor influencing the dissipation half-lives of TSPEOn in the cowpea ecosystem.

### Distribution of tristyrylphenol ethoxylates in cowpea ecosystem

**FIGURE 5:**
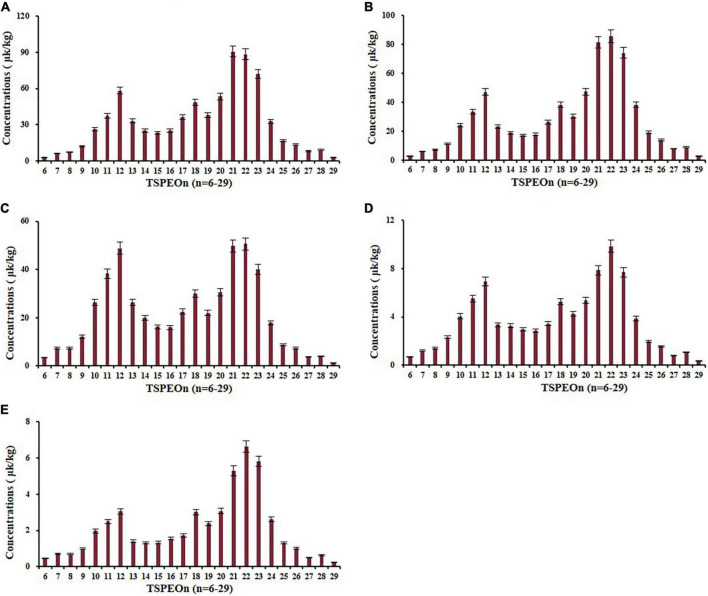
Concentration distribution of homolog tristyrylphenol ethoxylates (TSPEOn) (*n* = 6–29) under 450 g/ha after two applications at different interval to harvest in cowpea in terminal residue experiments in China. Interval to harvest: **(A)** 5 d; **(B)** 7 d; **(C)** 10 d; **(D)** 14 d; **(E)** 21 d.

The terminal residues of ΣTSPEOn in cowpea are shown in Supplementary Figure 1. The terminal concentrations of ΣTSPEOn were detected and ranged from 40.0 to 1,374 μg/kg in cowpea, which increased with the incremental application frequency and dosage. The typical distributions of homolog TSPEOn (*n* = 6–29) at 450 g/ha after two applications in cowpea in terminal residue experiments were characterized in Figure 5, and the distributions of other terminal residue experiments were shown in Supplementary Figures 2–4. It was found that a significant bimodal profile was observed in the homolog TSPEOn (*n* = 6–29) distribution in cowpea. One concentration peak-value was occurred at TSPEO12 (3.04–58.3 μg/kg), and the other was observed at TSPEO22 (6.22–88.4 μg/kg).

**FIGURE 6:**
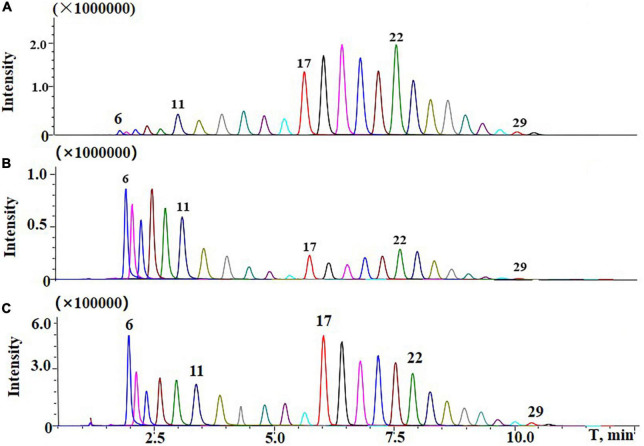
Typical liquid chromatography-tandem mass spectrometry (LC-MS/MS) chromatograms of **(A)** standard of commercial tristyrylphenol ethoxylates (TSPEO) homologs spiked at 1.0 mg/kg; **(B)** TSPEO homologs in cowpea under 450 g/ha after three applications at the interval to harvest of 10 d in terminal residues experiments; **(C)** TSPEO homologs in soil under 450 g/ha after three applications at the interval to harvest of 10 d in terminal residue experiments.

As shown in Figure 6, a typical normal distribution profile was presented in the commercial TSPEO mixture, but bimodal profiles were observed for TSPEOn in cowpea and soil samples. Compared with the commercial TSPEO mixture, the contributions of TSPEO homologs with short EO unites (*n* = 6–13) increased from 21.8 to 33.3% in cowpea and soil. All these results implied that the biotransformation would be taken place among the homologs TSPEOn (*n* = 6–29) in the cowpea ecosystem. However, it has been reported that the long-chain nonylphenol ethoxylate (NPEOn) can biodegraded into more lipophilic shortened EO chain NPEOn by attacking and shortening the hydrophilic part of the molecule of NPEOn under anaerobic conditions (31–34). Short-chain NPEOn presented more toxicity and persistence than long-chain nonylphenol ethoxylate (NPEOn).

### Risk assessment of tristyrylphenol ethoxylates in cowpea

Assessments of acute and chronic dietary intake risk for cowpea consumption are shown in Table 1. For the acute dietary intake risk, the HRs of ΣTSPEOn in cowpea samples were 1,374, 957, 560, 200, and 301 μg/kg at the interval to harvest of 5, 7, 10, 14, and 21 d, respectively. Accordingly, the aHI values for child (≤ 11 years), youngster (12–18 years), adult (18–60 years), and elder (> 60 years) were 0.04–0.30%, 0.03–0.19%, 0.02–0.15%, 0.02–0.15% for males, and 0.05–0.32%, 0.03–0.18%, 0.02–0.16%, 0.02–0.16% for females, respectively. These results indicate that there is little or no acute risk to humans.

**TABLE 1:** The acute and chronic dietary risk assessment of tristyrylphenol ethoxylates (TSPEOn) in cowpea.

Subgroup	Sex	Interval to harvest (days)	HR (μ g/kg)	NESTI (mg/kg bw/d)	aHI (%)	STMR (μ g/kg)	NEDI (mg/kg bw/d)	HQ (%)
Child	M	5	1,374	0.0050	0.30	770	0.0020	0.40
		7	957	0.0035	0.21	639	0.0017	0.33
		10	560	0.0020	0.12	320	0.0008	0.17
		14	200	0.0007	0.04	144	0.0004	0.08
		21	301	0.0011	0.07	103	0.0003	0.05
	F	5	1,374	0.0053	0.32	770	0.0020	0.40
		7	957	0.0037	0.22	639	0.0017	0.33
		10	560	0.0022	0.13	320	0.0008	0.17
		14	200	0.0008	0.05	144	0.0004	0.08
		21	301	0.0012	0.07	103	0.0003	0.05
Youngster	M	5	1,374	0.0031	0.19	770	0.0014	0.28
		7	957	0.0022	0.13	639	0.0012	0.23
		10	560	0.0013	0.08	320	0.0006	0.12
		14	200	0.0005	0.03	144	0.0003	0.05
		21	301	0.0007	0.04	103	0.0002	0.04
	F	5	1,374	0.0030	0.18	770	0.0014	0.27
		7	957	0.0021	0.13	639	0.0011	0.22
		10	560	0.0012	0.07	320	0.0006	0.11
		14	200	0.0004	0.03	144	0.0003	0.05
		21	301	0.0007	0.04	103	0.0002	0.04
Adult	M	5	1,374	0.0026	0.15	770	0.0012	0.24
		7	957	0.0018	0.11	639	0.0010	0.20
		10	560	0.0010	0.06	320	0.0005	0.10
		14	200	0.0004	0.02	144	0.0002	0.04
		21	301	0.0006	0.03	103	0.0002	0.03
	F	5	1,374	0.0026	0.16	770	0.0013	0.25
		7	957	0.0018	0.11	639	0.0010	0.21
		10	560	0.0011	0.06	320	0.0005	0.10
		14	200	0.0004	0.02	144	0.0002	0.05
		21	301	0.0006	0.03	103	0.0002	0.03
Elder	M	5	1,374	0.0024	0.15	770	0.0012	0.24
		7	957	0.0017	0.10	639	0.0010	0.20
		10	560	0.0010	0.06	320	0.0005	0.10
		14	200	0.0004	0.02	144	0.0002	0.05
		21	301	0.0005	0.03	103	0.0002	0.03
	F	5	1,374	0.0027	0.16	770	0.0013	0.25
		7	957	0.0019	0.11	639	0.0010	0.21
		10	560	0.0011	0.07	320	0.0005	0.10
		14	200	0.0004	0.02	144	0.0002	0.05
		21	301	0.0006	0.04	103	0.0002	0.03

For the chronic dietary intake risk, the STMRs of ΣTSPEOn in cowpea were 770, 639, 320, 144, and 103 μg/kg at the interval to harvest of 5, 7, 10, 14, and 21 d, respectively. Therefore, the HQs for child (≤ 11 years), youngster (12–18 years), adult (18–60 years), and elder (> 60 years) were 0.05–0.40%, 0.04–0.28%, 0.03–0.24%, and 0.03–0.24% for male, 0.05–0.40%, 0.04–0.27%, 0.03–0.25%, 0.03–0.25% for female, respectively, significantly lower than the acceptable risk level (100%). These results suggest that the risk of chronic dietary intake of ΣTSPEOn based on the terminal residues of different interval to harvest is acceptably low. The assessment results were coincided with the study of cucumber (10). Nevertheless, it should be noted that children are the most susceptible population to acute dietary intake risk and chronic dietary intake risk, and the impact on the health of children should be monitored in future.

## Conclusion

In the present study, the dissipation and terminal residues of TSPEO homologs in a cowpea ecosystem were studied. The dissipation rates of all the homolog TSPEOn (*n* = 6–29) in cowpea were higher than in soil. The long-chain TSPEOn presented a higher dissipation rate than that of short-chain TSPEOn in the cowpea ecosystem. The fact that the typical bimodal profiles of TSPEO homologs and the noticeable increase of short TSPEOn (*n* = 6–13) indicated that the long-chain TSPEOn would be degraded to short-chain TSPEOn in the cowpea ecosystem. The risks of acute and chronic dietary intake of ΣTSPEOn in cowpea for general consumers in China were distinctly lower than the acceptable levels (100%). But children were the most susceptible population to acute and chronic dietary intake risks, which should be paid more attention to. This study provides proper guidance and feasibility suggestions for the TSPEOn application in pesticide formulations.

## Data availability statement

The raw data supporting the conclusions of this article will be made available by the authors, without undue reservation.

## Author contributions

ML: investigation, sample processing, and writing – original draft. QW: formal analysis. XL: investigation. NY: sample processing. MJ: writing – review and editing. LZ: methodology and data curation. JW: supervision. FJ: writing – review and editing and funding acquisition. All authors contributed to the article and approved the submitted version.
