# Supplementary material for: Different dissipation potential and dietary risk assessment of tristyrylphenol ethoxylates in cowpea ecosystem in China
Source: Front Nutr. 2022 Oct 19;9:1036025. doi: 10.3389/fnut.2022.1036025 (PMC9626860; doi:10.3389/fnut.2022.1036025)
Supplement: Supplementary file 1 [file Data_Sheet_1.docx]

Supplementary Material

# Supplementary Figures and Tables

## Supplementary Figures


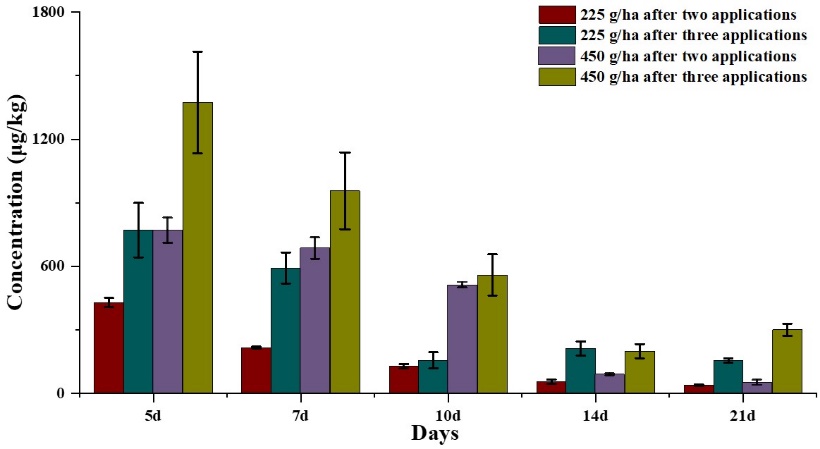


**Supplementary Figure 1.** Terminal residues of ∑TSPEOn in cowpea.


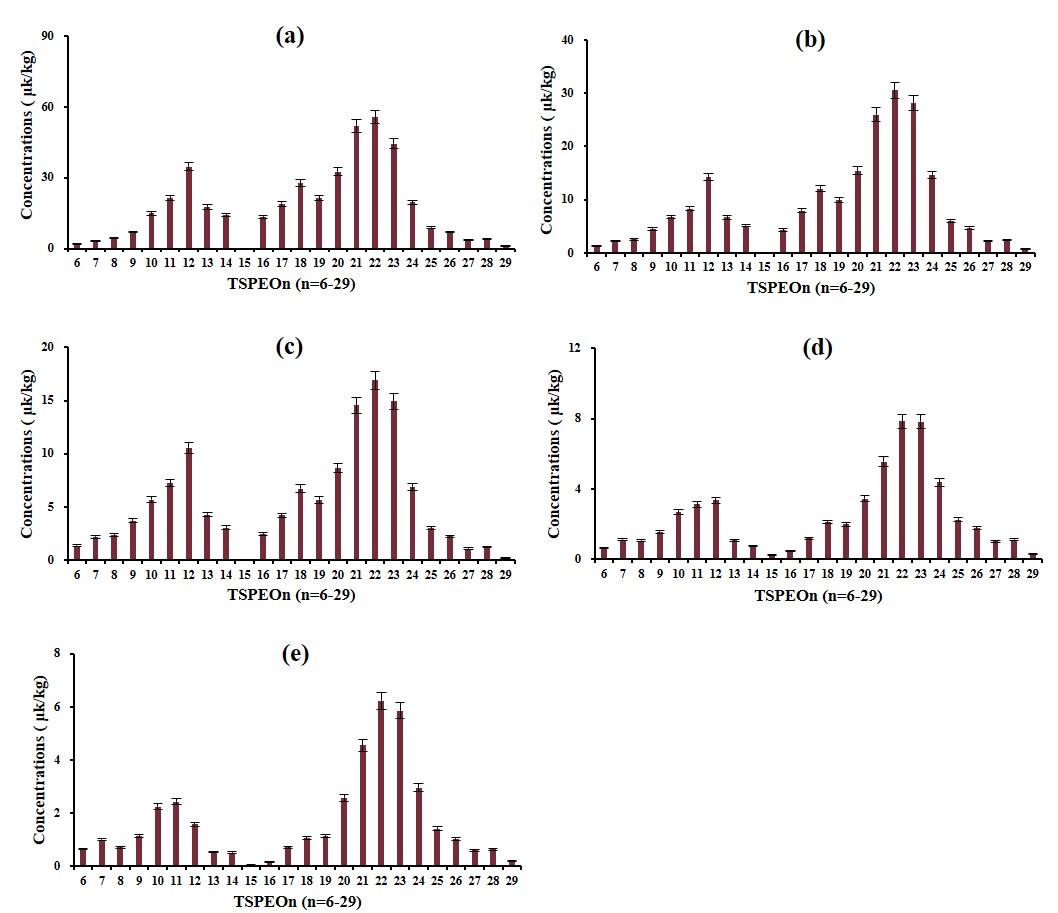


**Supplementary Figure 2.** Concentration distribution of homolog TSPEOn (n=6-29) under 225 g/ha after two applications at different intervals to harvest in cowpea in terminal residue experiments in China. Interval to harvest: (a) 5 d; (b) 7 d; (c) 10 d; (d) 14 d; (e) 21 d.


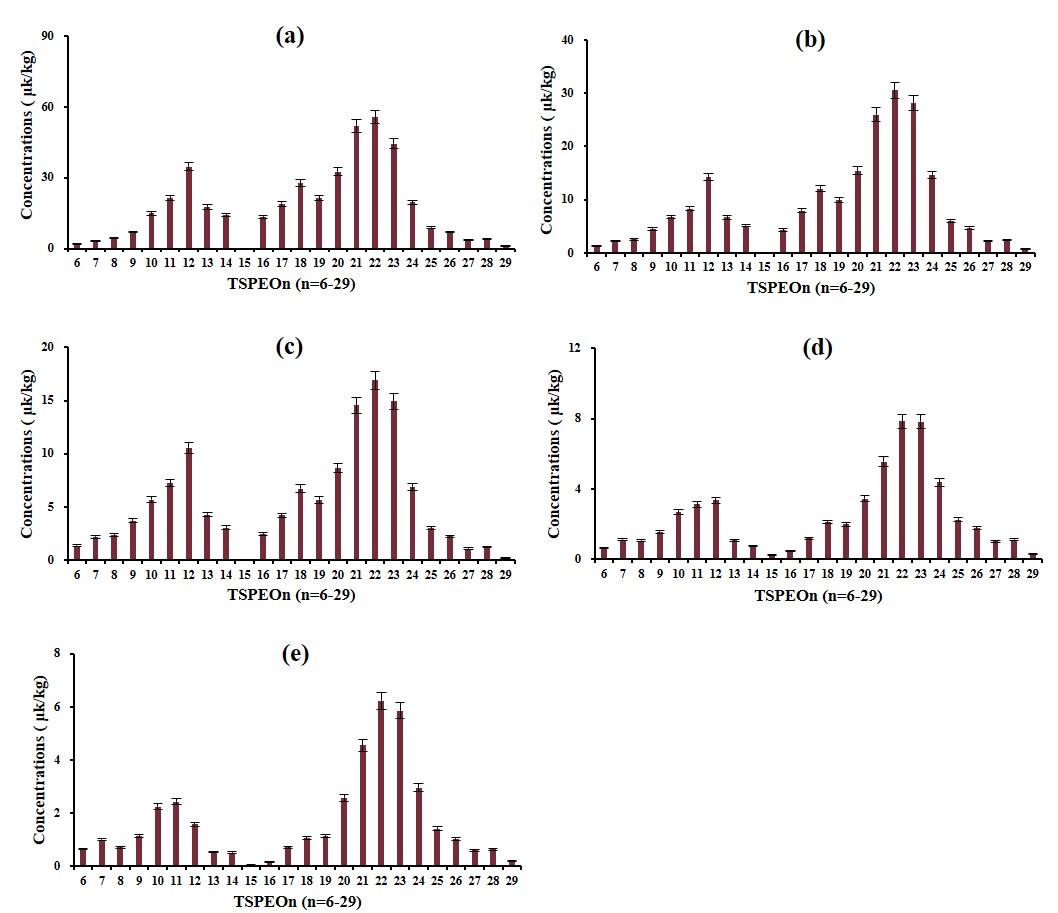


**Supplementary Figure 3.** Concentration distribution of homolog TSPEOn (n=6-29) under 225 g/ha after three applications at different intervals to harvest (PHI) in cowpea in terminal residue experiments in China. Interval to harvest: (a) 5 d; (b) 7 d; (c) 10 d; (d) 14 d; (e) 21 d.


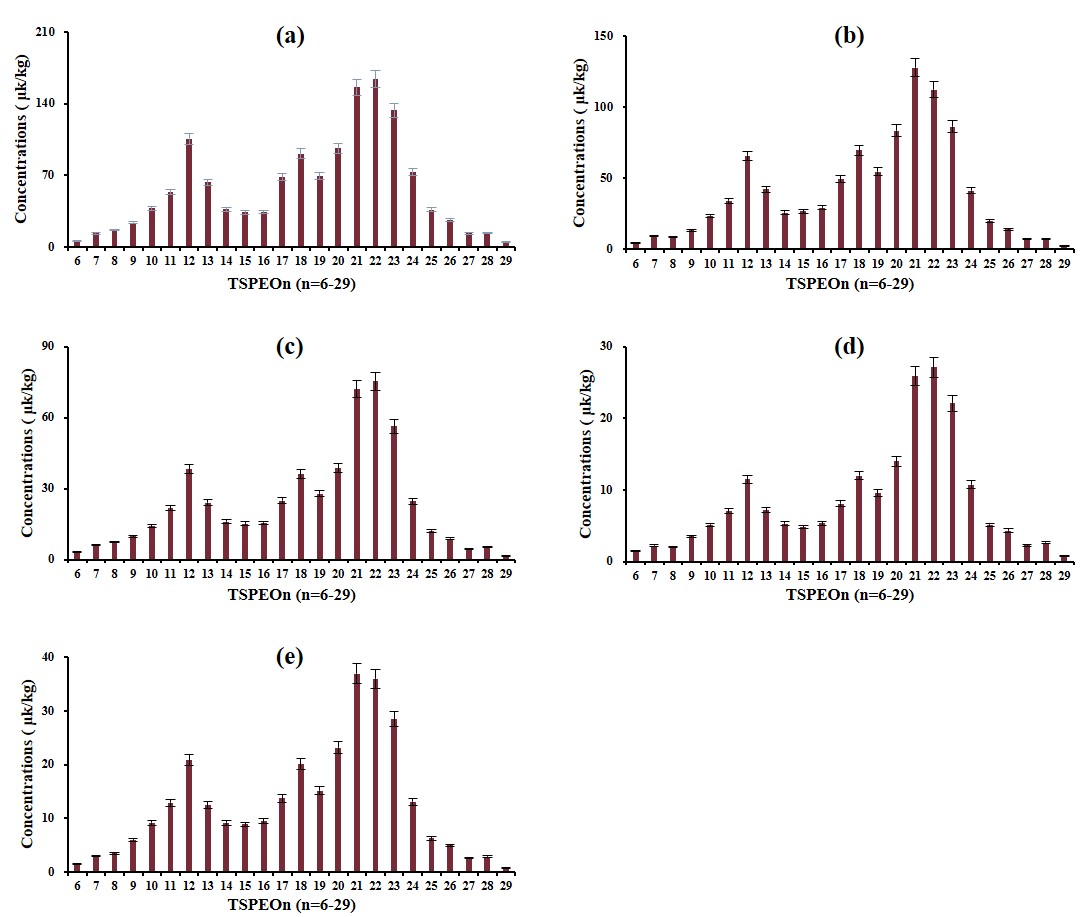


**Supplementary Figure 4.** Concentration distribution of homolog TSPEOn (n=6-29) under 450 g/ha after three applications at different intervals to harvest (PHI) in cowpea in terminal residue experiments in China. Interval to harvest: (a) 5 d; (b) 7 d; (c) 10 d; (d) 14 d; (e) 21 d.

## Supplementary Tables

**Table S1 Liquid chromatography-mass spectrometric parameters of 24 TSPEO homologs.**

| No. | Abbr. Name | Retention time (min) | Adduct ion | MRM transitions* | Q1 (V) | CE (V) | Q3 (V) |
| --- | --- | --- | --- | --- | --- | --- | --- |
| 1 | TSPEO6 | 1.968 | [M+NH_4_]^+^ | **688.5>105.2** | -35 | -63 | -44 |
|  |  |  |  | 688.5>567.4 | -35 | -20 | -41 |
| 2 | TSPEO7 | 2.145 | [M+NH_4_]^+^ | **732.5>105.1** | -35 | -65 | -17 |
|  |  |  |  | 732.5>89.2 | -35 | -37 | -35 |
| 3 | TSPEO8 | 2.378 | [M+NH_4_]^+^ | **776.6>133.10** | -29 | -33 | -47 |
|  |  |  |  | 776.6>551.4 | -29 | -25 | -38 |
| 4 | TSPEO9 | 2.666 | [M+NH_4_]^+^ | **820.6>105.1** | -32 | -60 | -41 |
|  |  |  |  | 820.6>329.2 | -41 | -30 | -14 |
| 5 | TSPEO10 | 3.046 | [M+NH_4_]^+^ | **864.7>105.1** | -44 | -65 | -41 |
|  |  |  |  | 864.7>89.1 | -26 | -38 | -14 |
| 6 | TSPEO11 | 3.498 | [M+NH_4_]^+^ | **908.7>105.2** | -11 | -59 | -17 |
|  |  |  |  | 908.7>177.0 | -35 | -31 | -10 |
| 7 | TSPEO12 | 3.923 | [M+NH_4_]^+^ | **952.7>105.1** | -38 | -65 | -23 |
|  |  |  |  | 952.7>89.0 | -50 | -46 | -14 |
| 8 | TSPEO13 | 4.305 | [M+NH_4_]^+^ | **996.7>105.1** | -38 | -65 | -17 |
|  |  |  |  | 996.7>329.3 | -29 | -36 | -20 |
| 9 | TSPEO14 | 4.703 | [M+NH_4_]^+^ | **1040.8>105.1** | -41 | -61 | -17 |
|  |  |  |  | 1040.8>89.3 | -32 | -51 | -38 |
| 10 | TSPEO15 | 5.136 | [M+NH_4_]^+^ | **1084.8>105.1** | -32 | -64 | -17 |
|  |  |  |  | 1084.8>89.1 | -32 | -45 | -35 |
| 11 | TSPEO16 | 5.532 | [M+NH_4_]^+^ | **1128.8>104.9** | -44 | -65 | -17 |
|  |  |  |  | 1128.8>133.1 | -44 | -38 | -47 |
| 12 | TSPEO17 | 5.887 | [M+2NH_4_]^2+^ | **595.6>105.2** | -30 | -22 | -42 |
|  |  |  |  | 595.6>89.2 | -30 | -29 | -34 |
| 13 | TSPEO18 | 6.266 | [M+2NH_4_]^2+^ | **617.6>105.2** | -24 | -23 | -16 |
|  |  |  |  | 617.6>89.2 | -24 | -31 | -36 |
| 14 | TSPEO19 | 6.64 | [M+2NH_4_]^2+^ | **639.6>105.2** | -12 | -26 | -32 |
|  |  |  |  | 639.6>89.2 | -48 | -31 | -14 |
| 15 | TSPEO20 | 6.91 | [M+2NH_4_]^2+^ | **661.7>105.2** | -26 | -40 | -40 |
|  |  |  |  | 661.7>89.2 | -34 | -33 | -34 |
| 16 | TSPEO21 | 7.198 | [M+2NH_4_]^2+^ | **683.7>105.2** | -26 | -38 | -14 |
|  |  |  |  | 683.7>89.2 | -26 | -33 | -34 |
| 17 | TSPEO22 | 7.524 | [M+2NH_4_]^2+^ | **705.7>105.2** | -12 | -37 | -40 |
|  |  |  |  | 705.7>89.2 | -36 | -34 | -32 |
| 18 | TSPEO23 | 7.85 | [M+2NH_4_]^2+^ | **727.7>105.1** | -28 | -44 | -16 |
|  |  |  |  | 727.7>89.2 | -28 | -35 | -34 |
| 19 | TSPEO24 | 8.169 | [M+2NH_4_]^2+^ | **749.8>105.1** | -38 | -55 | -16 |
|  |  |  |  | 749.8>89.2 | -28 | -35 | -14 |
| 20 | TSPEO25 | 8.54 | [M+2NH_4_]^2+^ | **771.8>105.1** | -38 | -48 | -16 |
|  |  |  |  | 771.8>89.2 | -38 | -36 | -36 |
| 21 | TSPEO26 | 8.882 | [M+2NH_4_]^2+^ | **793.8>105.1** | -30 | -52 | -16 |
|  |  |  |  | 793.8>89.2 | -30 | -36 | -34 |
| 22 | TSPEO27 | 9.205 | [M+2NH_4_]^2+^ | **815.9>105.1** | -42 | -54 | -16 |
|  |  |  |  | 815.9>89.2 | -32 | -37 | -14 |
| 23 | TSPEO28 | 9.556 | [M+2NH_4_]^2+^ | **837.9>105.2** | -32 | -49 | -41 |
|  |  |  |  | 837.9>89.2 | -32 | -38 | -20 |
| 24 | TSPEO29 | 9.901 | [M+2NH_4_]^2+^ | **859.9>105.1** | -32 | -50 | -41 |
|  |  |  |  | 859.9>89.2 | -32 | -42 | -35 |

*^a^* Note: * Quantifier transition in bold.

**Table S2 Method validation of 24 TSPEO homologs in cowpea and soil.**

| Analyte | Linear range (μg/kg) | Spiking level (μg/kg) | Cowpea | | | | | Coil | | | | |
| --- | --- | --- | --- | --- | --- | --- | --- | --- | --- | --- | --- | --- |
|  |  |  | R^2^ | Mean recoveries (%) | RSD (%) | LODs (μg/kg) | LOQs (μg/kg) | R^2^ | Mean recoveries (%) | RSD (%) | LODs (μg/kg) | LOQs (μg/kg) |
| TSPEO6 | 0.02-1.17 | 0.06 | 0.994 | 103 | 13.5 | 0.003 | 0.01 | 0.999 | 96.3 | 4.80 | 0.0002 | 0.001 |
|  |  | 0.12 |  | 90.2 | 6.50 |  |  |  | 84.6 | 11.1 |  |  |
|  |  | 0.58 |  | 97.9 | 5.40 |  |  |  | 100 | 7.10 |  |  |
| TSPEO7 | 0.06-3.12 | 0.16 | 0.994 | 97.9 | 12.9 | 0.01 | 0.04 | 0.993 | 95.5 | 11.7 | 0.001 | 0.002 |
|  |  | 0.31 |  | 93.8 | 4.00 |  |  |  | 95.7 | 14.2 |  |  |
|  |  | 1.56 |  | 98.3 | 3.30 |  |  |  | 90.0 | 4.90 |  |  |
| TSPEO8 | 0.14-7.09 | 0.35 | 0.996 | 100 | 12.2 | 0.03 | 0.09 | 0.999 | 79.3 | 6.10 | 0.002 | 0.005 |
|  |  | 0.71 |  | 108 | 3.90 |  |  |  | 83.5 | 7.60 |  |  |
|  |  | 3.54 |  | 97.2 | 11.1 |  |  |  | 83.5 | 3.40 |  |  |
| TSPEO9 | 0.28-14.1 | 0.70 | 0.996 | 111 | 6.50 | 0.01 | 0.04 | 0.998 | 87.6 | 13.3 | 0.002 | 0.006 |
|  |  | 1.41 |  | 103 | 6.80 |  |  |  | 113 | 3.70 |  |  |
|  |  | 7.03 |  | 106 | 7.10 |  |  |  | 69.6 | 7.30 |  |  |
| TSPEO10 | 0.49-24.7 | 1.24 | 0.996 | 82.5 | 3.90 | 0.01 | 0.04 | 0.998 | 91.2 | 3.90 | 0.003 | 0.01 |
|  |  | 2.47 |  | 81.0 | 10.6 |  |  |  | 99.0 | 2.50 |  |  |
|  |  | 12.4 |  | 92.2 | 3.70 |  |  |  | 75.0 | 7.20 |  |  |
| TSPEO11 | 0.78-39.0 | 1.95 | 0.997 | 90.6 | 13.6 | 0.04 | 0.15 | 0.999 | 86.7 | 13.3 | 0.004 | 0.01 |
|  |  | 3.90 |  | 110 | 10.3 |  |  |  | 88.6 | 5.70 |  |  |
|  |  | 19.5 |  | 83.4 | 6.50 |  |  |  | 82.5 | 12.9 |  |  |
| TSPEO12 | 1.12-55.8 | 2.79 | 0.998 | 92.0 | 14.2 | 0.06 | 0.20 | 0.999 | 86.5 | 12.8 | 0.007 | 0.02 |
|  |  | 5.58 |  | 82.7 | 18.1 |  |  |  | 82.9 | 8.70 |  |  |
|  |  | 27.9 |  | 92.0 | 4.40 |  |  |  | 85.5 | 10.7 |  |  |
| TSPEO13 | 1.46-73.1 | 3.65 | 0.999 | 89.0 | 14.7 | 0.10 | 0.32 | 0.999 | 98.4 | 3.20 | 0.009 | 0.03 |
|  |  | 7.31 |  | 103 | 12.3 |  |  |  | 79.8 | 11.8 |  |  |
|  |  | 36.5 |  | 103 | 6.00 |  |  |  | 84.9 | 4.10 |  |  |
| TSPEO14 | 1.76-88.1 | 4.40 | 0.996 | 102 | 15.0 | 0.10 | 0.32 | 0.998 | 90.6 | 12.3 | 0.01 | 0.04 |
|  |  | 8.81 |  | 120 | 14.1 |  |  |  | 85.1 | 9.00 |  |  |
|  |  | 44.0 |  | 90.6 | 5.60 |  |  |  | 78.0 | 8.60 |  |  |
| TSPEO15 | 1.97-98.4 | 4.92 | 0.999 | 97.5 | 16.9 | 0.14 | 0.48 | 0.999 | 95.2 | 14.4 | 0.01 | 0.04 |
|  |  | 9.84 |  | 85.9 | 19.1 |  |  |  | 81.3 | 7.80 |  |  |
|  |  | 49.2 |  | 91.8 | 3.10 |  |  |  | 79.3 | 16.4 |  |  |
| TSPEO16 | 2.05-102 | 5.13 | 0.999 | 95.1 | 16.6 | 0.11 | 0.36 | 0.997 | 92.7 | 17.3 | 0.01 | 0.04 |
|  |  | 10.2 |  | 115 | 3.70 |  |  |  | 88.9 | 7.00 |  |  |
|  |  | 51.2 |  | 104 | 7.30 |  |  |  | 82.9 | 10.9 |  |  |
| TSPEO17 | 2.00-99.9 | 5.00 | 0.997 | 103 | 8.10 | 0.11 | 0.36 | 0.999 | 93.1 | 10.0 | 0.01 | 0.03 |
|  |  | 9.99 |  | 103 | 7.60 |  |  |  | 96.2 | 6.60 |  |  |
|  |  | 50.0 |  | 84.1 | 0.90 |  |  |  | 88.8 | 8.40 |  |  |
| TSPEO18 | 1.83-91.5 | 4.58 | 0.999 | 117 | 12.1 | 0.02 | 0.07 | 0.998 | 90.3 | 12.4 | 0.01 | 0.04 |
|  |  | 9.15 |  | 109 | 14.1 |  |  |  | 82.4 | 5.60 |  |  |
|  |  | 45.8 |  | 88.3 | 4.40 |  |  |  | 83.4 | 9.00 |  |  |
| TSPEO19 | 1.58-79.1 | 3.95 | 0.995 | 118 | 9.00 | 0.02 | 0.05 | 0.997 | 90.3 | 6.50 | 0.01 | 0.04 |
|  |  | 7.91 |  | 88.2 | 5.50 |  |  |  | 82.4 | 3.60 |  |  |
|  |  | 39.5 |  | 93.0 | 5.20 |  |  |  | 83.4 | 6.90 |  |  |
| TSPEO20 | 1.29-64.6 | 3.23 | 0.992 | 111 | 15.6 | 0.02 | 0.07 | 0.992 | 77.4 | 9.00 | 0.01 | 0.04 |
|  |  | 6.46 |  | 96.2 | 18.8 |  |  |  | 94.0 | 7.40 |  |  |
|  |  | 32.3 |  | 97.9 | 9.30 |  |  |  | 93.1 | 4.40 |  |  |
| TSPEO21 | 1.00-50.1 | 2.51 | 0.995 | 99.1 | 20.1 | 0.01 | 0.04 | 0.998 | 95.7 | 6.30 | 0.01 | 0.03 |
|  |  | 5.01 |  | 97.6 | 15.1 |  |  |  | 81.5 | 12.2 |  |  |
|  |  | 25.1 |  | 100 | 6.80 |  |  |  | 92.5 | 16.0 |  |  |
| TSPEO22 | 0.74-37.0 | 1.85 | 0.996 | 106 | 7.10 | 0.10 | 0.30 | 0.998 | 82.7 | 7.10 | 0.01 | 0.02 |
|  |  | 3.70 |  | 93.4 | 16.4 |  |  |  | 88.2 | 12.7 |  |  |
|  |  | 18.5 |  | 96.2 | 5.70 |  |  |  | 83.2 | 14.2 |  |  |
| TSPEO23 | 0.52-26.0 | 1.30 | 0.998 | 96.9 | 19.2 | 0.10 | 0.02 | 0.994 | 76.4 | 14.0 | 0.003 | 0.02 |
|  |  | 2.60 |  | 101 | 12.7 |  |  |  | 66.1 | 8.40 |  |  |
|  |  | 13.0 |  | 105 | 8.10 |  |  |  | 93.2 | 8.50 |  |  |
| TSPEO24 | 0.35-17.5 | 0.88 | 0.998 | 93.1 | 15.7 | 0.004 | 0.01 | 0.996 | 86.3 | 10.9 | 0.01 | 0.01 |
|  |  | 1.75 |  | 90.8 | 9.90 |  |  |  | 79.4 | 4.70 |  |  |
|  |  | 8.75 |  | 104 | 3.50 |  |  |  | 78.1 | 8.80 |  |  |
| TSPEO25 | 0.23-11.3 | 0.56 | 0.990 | 91.0 | 14.3 | 0.003 | 0.01 | 0.997 | 77.8 | 9.00 | 0.01 | 0.03 |
|  |  | 1.13 |  | 79.7 | 11.4 |  |  |  | 79.8 | 9.80 |  |  |
|  |  | 5.63 |  | 97.4 | 1.10 |  |  |  | 82.2 | 13.0 |  |  |
| TSPEO26 | 0.14-7.00 | 0.35 | 0.995 | 99.3 | 8.20 | 0.002 | 0.01 | 0.998 | 79.9 | 9.90 | 0.01 | 0.03 |
|  |  | 0.70 |  | 89.4 | 10.9 |  |  |  | 89.5 | 7.50 |  |  |
|  |  | 3.48 |  | 98.0 | 2.20 |  |  |  | 77.4 | 3.80 |  |  |
| TSPEO27 | 0.08-4.13 | 0.21 | 0.990 | 84.5 | 13.9 | 0.003 | 0.01 | 0.996 | 73.5 | 8.40 | 0.01 | 0.03 |
|  |  | 0.41 |  | 110 | 12.3 |  |  |  | 77.2 | 7.50 |  |  |
|  |  | 2.06 |  | 98.1 | 1.70 |  |  |  | 64.2 | 10.5 |  |  |
| TSPEO28 | 0.05-2.36 | 0.12 | 0.994 | 101 | 6.60 | 0.002 | 0.10 | 0.999 | 77.1 | 8.80 | 0.01 | 0.03 |
|  |  | 0.24 |  | 112 | 8.80 |  |  |  | 83.4 | 10.3 |  |  |
|  |  | 1.18 |  | 100 | 0.70 |  |  |  | 89.6 | 6.40 |  |  |
| TSPEO29 | 0.03-1.30 | 0.07 | 0.998 | 92.6 | 12.0 | 0.001 | 0.004 | 0.991 | NA | NA | 0.01 | 0.05 |
|  |  | 0.13 |  | 104 | 0.70 |  |  |  | 74.5 | 1.30 |  |  |
|  |  | 0.65 |  | 102 | 3.20 |  |  |  | 67.2 | 11.0 |  |  |

*^a^* Note: NA, data are not available.

**Table S3 Body weights and consumption data of cowpea for the eight subgroups.**

| Subgroup | Sex | Body weight (kg) | LP (P97.5)  (g/day person) | F (mean)  (g/day person) |
| --- | --- | --- | --- | --- |
| Child | M | 21.2 | 77.3 | 55.3 |
|  | F | 20.4 | 79.2 | 53.2 |
| Youngster | M | 45.9 | 104 | 84.0 |
|  | F | 43.9 | 96.6 | 77.2 |
| Adult | M | 62.5 | 117 | 95.7 |
|  | F | 55.1 | 106 | 90.0 |
| Elder | M | 58.5 | 104 | 92.5 |
|  | F | 50.8 | 99.6 | 83.3 |
